# A comparative cross-sectional study of prevalence and intensity of soil-transmitted helminthic infection between healthy and severe acutely malnourished pre-school aged children in Kano, Northern Nigeria

**DOI:** 10.1186/s12879-019-3755-3

**Published:** 2019-02-06

**Authors:** Abdulazeez Imam, Zubaida L. Farouk, Fatimah Hassan-Hanga, Uchechukwu G. Ihesiulor

**Affiliations:** 1 Department of Vaccines and Immunity, Medical Research Council Unit The Gambia at London school of Hygiene and Tropical Medicine, Atlantic Boulevard, P.O. Box 452, Fajara, Gambia; 2 Department of Paediatrics, Bayero University Kano, Kano, Nigeria; 3 Department of Paediatrics, Aminu Kano Teaching Hospital, Kano, Nigeria; 4 Department of Medical Microbiology and Parasitology, Bayero University Kano, Kano, Nigeria

**Keywords:** Soil-transmitted helminthic infections, Severe acute malnutrition, Intensity of infection, Pre-school children

## Abstract

**Background:**

Soil-transmitted helminthic (STH) infections are common in Sub-Saharan Africa. One method used for control of these helminths is mass anti-helminthic administration in populations at risk of STH infections. In this regard, empiric treatment of children with Severe Acute Malnutrition (SAM) for STH infection is practiced in this region. It is however unclear if children with SAM suffer more from STH infection than healthy children. The objective of this study was to compare prevalence and intensity of STH infection between pre-school aged children with SAM and healthy children.

**Methods:**

We approached 1114 pre-school aged children attending care in two health facilities in Kano, Nigeria to partake in this study. Of this number, we recruited 620 (55.7%) children, comprising 310 well-nourished children from well-baby clinics and 310 children with SAM from Community Management for Acute Malnutrition (CMAM) centres in these facilities. We assessed their nutritional status using World Health Organisation (WHO) growth charts and collected stool samples which we analysed using Formal-Ether Concentration technique to identify STH infection and Stoll’s technique to assess intensities of STH infection. We fitted a logistic regression model to determine if there was any association between nutrition status and helminthic infection, adjusting for the confounding effects of socio-economic status and age. We compared intensity of STH infection (measured as eggs per gram of faeces) between both nutrition groups using the independent t-test.

**Results:**

Overall STH prevalence in our population was low (2.7%) and we found no significant association between nutritional status and presence of STH infection (OR = 1.10, 95% CI 0.38 to 3.21). Majority of our study participants had either low or moderate (94.2%) and there was no statistically significant difference between intensity of STH infection (t value = − 1.52, *P* value = 0.13) in children with SAM and those who were well-nourished.

**Conclusions:**

The overall STH prevalence among pre-school children was low in Kano and we did not find prevalence and intensity of STH infection to differ significantly between preschool children with SAM and well-nourished children. Our findings confirm the WHO recommendation that at low levels of prevalence and intensity, interventions to control STH are unnecessary.

## Background

Soil-transmitted helminths (STH) is a collective term for a group of worms that cause infection in humans, when they come in contact with parasitic eggs or larvae which thrive in soil [1]. Three of these worms, ascaris, trichuiris and hookworm are of the greatest public health significance and infections with these helminths are widely distributed throughout the tropics and subtropics; where the climate, poverty, inadequate water supply and sanitation are important determinants of transmission [1]. Morbidity from these helminths and their rates of transmission are directly related to the number of worms harbored by the host and this is referred to as the intensity of infection [2]. The intensity of STH infection is measured by the number of eggs per gram of faeces and is the main epidemiological index used to describe STH infection [3].

Globally, an estimated 850 million children are infected with at least one STH, and a large proportion of these infections occur in Sub-Saharan Africa (SSA) where all countries in the region are thought to be endemic [4, 5]. In Nigeria, STH infection is endemic and prevalence is estimated to be 13.8% in school-aged children [6]. These helminths have been associated with severe anaemia, pronounced protein loss, micronutrient deficiency, and in the long term, impaired physical, intellectual and cognitive development, as well as malnutrition, especially in children with higher intensities of infection [7–10].

Strategies for global STH infection control include periodic mass drug administration, particularly in regions were these worms are prevalent or in populations that are at high risk for STH infection [11]. In this regard, the World Health Organization (WHO) recommends empiric treatment of STH infection in children with Severe Acute Malnutrition (SAM) as part of community management of this condition in areas that are endemic for STH [12, 13]. The rationale behind these mass drug treatments is interruption of STH transmission which eventually leads to reduced population worm burden and a reduction in helminth-associated morbidity [14]. Globally however, scientific evidence relating STH infections to childhood nutritional status has largely arisen from studies demonstrating decreased anthropometric parameters in helminth-infected children, or those that have described improvements in these parameters following anti-helminthics administration to these children [8, 15–17]. Less is known to what extent nutritional status might affect host susceptibility to infection with these parasites [18]. More so, mass anti-helminthic drug administration, particularly if not needed might be associated with an increased risk for development of anti-helminthic resistance in humans [19–21]. This study thus compared prevalence and intensity of STH infection between a group of severely acutely malnourished pre-school children and their well-nourished (healthy) counterparts in a setting presumably endemic for both STH infection and malnutrition.

## Methods

### Study setting

The study was conducted in Kano state, North-Western Nigeria between November 2016 and May 2017. The local climate is semi-arid and typically hot all year round with average temperatures of 24.6 °C, and savannah-type vegetation. Kano is endemic for STH children particularly among school-age children [6, 22]. About 1.1% of children between the ages of 6 and 59 months in the state have Severe Acute Malnutrition (SAM) [23]. In the absence of medical complications or nutritional oedema, these children are treated with Ready to Use Therapeutic Foods (RUTF) at Community Management for Acute Malnutrition (CMAM) Out-patient Therapeutic Programs (OTPs). The CMAM program run by United Nations Children’s Fund (UNICEF) in collaboration with the Nigerian Federal Ministry of Health is a community based integrated approach that aims to improve coverage for the management of SAM [24]. Children seen in OTPs come from communities surrounding the treatment centres. Kano state has 30 of such centres spread across 6 Local Government Areas. Two of these OTPs located in Murtala Muhammed Specialist Hospital and Ali Akilu specialist hospital were selected for recruitment of study participants using a multi-stage sampling technique.

### Study population

The study population consisted of two groups: A ‘healthy’ group comprising well-nourished children and a group who had SAM. The SAM group were new admissions into the CMAM program at the study sites, while the well-nourished group were recruited from immunisation/well-baby clinics and out-patient clinics in the same facilities. Participant nutritional status was defined using WHO reference weight-for height z-score charts [24]. Using WHO z-score thresholds, children with weight-for-height z-scores < − 3 SD were categorised as having SAM while those with z-scores ≥ − 2 SD were classified as well-nourished [24].

### Study design

This was an analytic cross-sectional study which compared the prevalence and intensity of soil-transmitted helminthic infection between pre-school children with SAM and their well-nourished counterparts.

### Sample size considerations

The sample size of the study was 620 children comprising 310 children with SAM and 310 well-nourished children. This was determined using the minimum number per group of children required for the study using the standard formula for sample size in a comparative study and setting the study power at 90% [25]. To derive our sample size we used prevalence data from a previously published local study on prevalence of STH in malnourished under-five children [26].

### Data collection

Subsequent on obtaining written informed consent from the parent/guardian, prospective study participants aged between 6 and 59 months were recruited during clinic visits. Each of these participants had relevant clinical and sociodemographic data entered in a specially designed questionnaire. Their lengths/heights and weights were measured and recorded using standard procedures [27]. The resulting measurements were then plotted on WHO standard growth charts to determine individual z-score values [28]. Children who were found to have Moderate Acute Malnutrition (MAM) (defined as z-score < − 2 but > − 3 using UNICEF criteria) or who had taken an anti-helminthic in the 6 months prior to the study were excluded.

### Stool collection and transport procedure

The parents of the study participants were properly instructed on stool sample collection. They were provided with labelled stool specimen containers and were asked to collect fresh early morning stool samples from their wards on the day of a scheduled follow-up visit to the OTP. Collected stool specimens were retrieved from parents and pooled in ice boxes and subsequently transported to the research lab.

### Stool sample processing

On arrival at the laboratory, an initial stool macroscopy was performed by the laboratory research team, following which an aliquot of 10% formal saline was added to the specimens for sample preservation and these were then refrigerated at 4 °C. Stool samples collected throughout the week were pooled for processing over the weekends. The Formol-Ether Concentration technique was used for stool processing for microscopy while the Stoll’s technique was used to estimate stool intensity of infection [29].

### Laboratory quality control measures

The Laboratory research team were blinded to the nutritional arm of the study participants during stool analysis and a random 10% of stool samples were double read by a second laboratory scientist independent of the research project.

### Data analysis

Data on socioeconomic status (measured as social class) was derived indirectly using a previous method described by Oyedeji et al [30]. The intensity of STH infection was classified into low, moderate and heavy intensities of infections using thresholds set by the World Health Organisation (WHO) [3].

For Univariate analysis comparing baseline demographic data across nutritional groups (SAM and well-nourished groups), the Mann-Whitney U test was used for variables that were continuous with skewed distribution while the chi-square test was used for categorical variables.

The prevalence of STH infection was determined by dividing the total number of positive stool samples by the total number of study participants. Helminthic subgroup prevalence was also determined for both SAM and well-nourished groups in a similar manner. The chi-square test was used to observe for any statistical significant differences in prevalence between the two groups. To adjust for the confounding effects of age and socioeconomic status, a logistic regression model was fitted to compare the prevalence of helminths between the nutrition groups. An initial base model (Univariate model) was run with nutritional status as a single covariate and subsequent models were adjusted for socioeconomic status and participant’s age. The best fit model was determined using Akaike’s Information Criteria (AIC). A lower AIC suggested a better fitting model. Model co-efficients were exponentiated to derive odds ratios and corresponding 95% confidence intervals.

The independent t-test was used to compare mean intensities of STH infection (measured as mean number of eggs per gram of stool) between both nutrition groups.

Statistical analysis was performed using STATA statistical software package version 13.

## Results

**Fig. 1:**
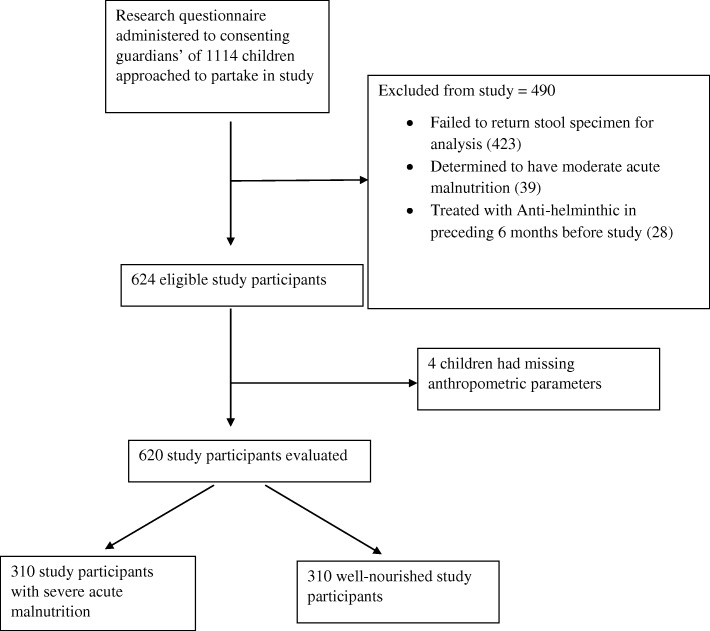
Consort diagram depicting reasons for research participant’s exclusion from study

Between November of 2016 and May of 2017, a total of 1114 children aged between 6 to 59 months were approached to participate in this study. Of this number, 620 (55.7%) children were included in the study as they met all inclusion criteria while 494 (44.3%) were excluded from the study. Figure 1 depicts the reasons for study participant exclusion.

There were 310 well-nourished children and 310 children with SAM. All children with SAM had marasmus and there were no study participants with either Kwashiorkor or Marasmic-Kwashiorkor. Median age ± interquartile range (IQR) of the study population was 14.0 ± 4.0 months. Median age ± IQR for the SAM group was 12.0 ± 9.0 while this was 18.0 ± 26.0 for the well-nourished group. There were 330 males (53.2%) and 290 females (46.8%).

Table 1 shows family and household characteristics for the study population across SAM and well-nourished groups. The proportion of parents who were not formally educated was significantly higher in the SAM group when compared to the well-nourished group. Both groups also differed in social class, as greater than 90% of the SAM group were in the lowest social classes (social class IV and V) as compared to about 65% of the well-nourished group. A larger proportion of the SAM group also came from families who were polygamous and had significantly larger number of people per household when compared to the well-nourished group. Median weekly household expenditure on perishable foods was also significantly lower in the SAM group. There was however no significant difference in the proportion of participant’s mothers who were employed between the groups.

**Table 1:** Family and Household characteristics compared between severely acutely malnourished (SAM) and well-nourished study population (*n* = 620)

Variable	Malnourished children (%) *n* = 310	Well-nourished children (%) n = 310	*P*-value	Total (%) n = 620
Family characteristics				
Paternal educational status				
Not formally educated	151 (48.7)	93 (30.0)	< 0.0001*	244 (39.4)
Formally educated	159 (51.3)	217 (70.0)		376 (60.6)
Maternal educational status				
Not formally educated	211 (41.9)	130 (68.1)	< 0.0001*	341 (55.0)
Formally educated	180 (58.1)	99 (31.9)		279 (45.0)
Maternal employment status				
Unemployed	90 (29.0)	105 (33.9)	0.19	195 (31.5)
employed	220 (71.0)	205 (66.1)		425 (68.5)
Family type^#^				
monogamous	111 (35.8)	148 (48.2)	0.002*	259 (42.0)
polygamous	199 (64.2)	159 (51.8)		358 (58.0)
Social class				
I	0 (0.0)	2 (0.7)	< 0.0001*	2 (0.3)
II	10 (3.2)	27 (8.7)		37 (6.0)
III	18 (5.8)	77 (24.8)		95 (15.3)
IV	140 (45.2)	129 (41.6)		269 (43.4)
V	142 (45.8)	75 (24.2)		217 (35.0)
Household characteristics	Median (IQR)	Median (IQR)	P-value	Range
Persons per household	7 (6)	9 (6)	0.02	3 33
Children per household	5 (5)	6 (5)	0.25	1 28
Weekly family expenditure on household perishable foods per person ^b^ (Nigerian Naira, *n* = 306) ^**^	214.8 (268.9)	291.7 (350)	0.0002*	15.42333.3

*-Statistically significant at *P* < 0.05, χ^2^ Chi-square, *FE* Fischer’s exact test

The prevalence of helminths among the study population was 2.7% (17/620). Of the total helminths isolated, 10 were from children with SAM giving a prevalence of 3.2% (10/310) in this group. The remaining 7 were from well-nourished children giving a prevalence of 2.3% (7/310). The most commonly isolated helminth was Ascaris and this accounted for 70.5% (12/17) of isolated helminths. Hookworm and Trichuris accounted for 23.5% (4/17) and 6% (1/17) of isolated helminths respectively. There were no significant differences in prevalence between SAM and well-nourished children. As shown in Table 2.

**Table 2:** Comparison of prevalence of soil-transmitted helminth infection among SAM and well-nourished children (n = 620)

Variable	Malnourished children (%)	Well-nourished children (%)	*P*-value	Total (%)
All helminths				
Absent	300 (96.8)	303 (97.7)	0.46^*^	603(97.3)
Present	10 (3.2)	7 (2.3)		17 (2.7)
Roundworm				
Absent	304 (98.1)	304 (98.1)	1.00^*^	608 (98.1)
Present	6 (1.9)	6 (1.9)		12 (1.9)
Hookworm				
Absent	307 (99.0)	309 (99.7)	0.62^*^	616 (99.4)
Present	3 (1.0)	1 (0.3)		4 (0.7)
Whipworm				
Absent	309 (99.7)	310 (100.0)	1.00^*^	619 (99.8)
Present	1 (0.3)	0 (0.0)		1 (0.2)

^*^- Not statistically significant at P < 0.05, χ^2^- Chisquare, *FE* Fischer’s exact test

Table 3 shows odd ratios and 95% confidence interval for the crude logistic regression models, best fitting multivariate model and an age and socioeconomic status adjusted model used to determine the association of nutritional status with helminthic infection. The best fitting model for the all helminth group involved adjusting for age alone, while for the Hookworm group this involved an adjustment for age and socioeconomic status. The crude model was the best fit model for Roundworm. Being malnourished was associated with a 10% increased odd of being infected by a soil transmitted helminth. This association was however not statistically significant (OR = 1.10, 95% CI 0.38 to 3.21). Being malnourished was also associated with a non-significant increased risk for infection with Hookworms (OR = 3.53, 95% CI 0.32 to 38.83). There was however no difference in the odds of being infected with Roundworm between malnourished and healthy children (OR = 1.00, 95% CI 0.32 to 3.14).

**Table 3:** Logistic regression models to determine association of nutritional status with helminthic infections

Helminthic groups	Univariate model^c^	Multivariate model^d^	Age and socioeconomic status adjusted model^e^
	Odds ratio (95% CI)	Odds ratio (95% CI)	Odds ratio (95% CI)
All helminths Combined^f^			
Malnourished	1.44 (0.54 3.84)	1.10^bg^ (0.38 3.21)	1.33 (0.44 4.07)
Well-nourished	1.00	1.00	1.00
Roundworm			
Malnourished	1.00^g^ (0.32 3.14)	0.83^b^ (0.24 2.91)	0.84 (0.23 3.06)
Well-nourished	1.00	1.00	1.00
Hookworm			
Malnourished	3.02 (0.31 29.19)	3.53^ag^ (0.32 38.83)	3.53^a^ (0.32 38.83)
Well-nourished	1.00	1.00	1.00

*CI* Confidence interval

^a^-Model adjusted for age and socioeconomic status

^b^- Model adjusted for age alone

^c^- Univariate models are crude logistic models with only study participants’ nutritional status as a single covariate

^d^- Multivariate models are best fitting adjusted logistic regression models (Models were adjusted for age, socioeconomic status or both covariates and the model with the best fit statistic was reported)

^e^- Age and socioeconomic status adjusted model are models adjusted for both age and socioeconomic status irrespective of fit statistics

^f^- All helminth combined group consists of Roundworm, Hookworm and Whipworm

^g^- Best fitting model

In the SAM group, mean (SD) the intensity of STH infection measured as mean eggs per gram of stool was 1.26 (1.3), while in the well-nourished group this was 1.11 (0.9). There was no statistically significant difference between both groups (t value = − 1.52, *P* value = 0.13).

Table 4 classifies all study participants into light, moderate or heavy intensities of STH infection.(3) Overall study participants had either light or moderate intensities of infection (47.1% for both categories). One of 17 (5.9%) had heavy intensities of infection. This participant had Hookworm infestation.

**Table 4:** Intensities of STH infection using World Health Organisation (WHO) Threshold

STH intensity of infection	Type of STH infection			
	Hookworm(%) *n* = 4	Ascaris(%) *n* = 12	Trichuris(%) n = 1	All STH^d^ (%) *n* = 17
Light^a^	1 (25.0)	7 (58.3)	0 (0.0)	8 (47.1)
Moderate^b^	2 (50.0)	5 (41.7)	1 (100.0)	8 (47.1)
Heavy^c^	1 (25.0)	0 (0.0)	0 (0.0)	1 (5.9)

*STH* Soil-transmitted helminth

^a^- Light intensity of infection for Ascaris was defined as egg per gram (EPG) of faeces between 1 and 4999, for Hookworm it is between 1 and 999 EPGs and for Trichuris 1-1999EPGs

^b^- Moderate intensity of infection for Ascaris was defined as egg per gram (EPG) of faeces between 5000 and 49,999, for Hookworm it is between 1000 and 9999 EPGs and for Trichuris 2000-3999EPGs

^c^- Heavy intensity of infection for Ascaris was defined as egg per gram (EPG) of faeces > 50,000, for Hookworm it is > 10,000 EPGs and for Trichuris > 4000 EPGs

^d^- All STH groups combine intensities of infection of Hookworm, Trichuris and Ascaris

## Discussion

Our study found no difference in the prevalence and the intensity of STH infections between children with SAM and healthy children. Overall helminth prevalence among the study participants was low and the majority had either low or moderate intensities of STH infection.

Our findings are similar to previous studies from Nigeria and the Philippines that have documented no significant differences in prevalence of STH infections between malnourished and well-nourished children [18, 31, 32]. By contrast, two other Nigerian studies reported malnourished children to have higher STH prevalence when compared to healthy children [26, 33], while a study among Zairean children reported a lower prevalence in children with SAM [34]. These observed differences might be due to differences in our inclusion criteria. While our malnourished cohort consisted only of children with SAM, both Nigerian studies investigated malnourished groups who had varying degrees of malnutrition [26, 33]. Interactions between STH infection and the malnourished host might depend on the hosts’ degree of malnutrition which in turn influences helminth acquisition. Bundy and Golden [35] suggest mild and moderate malnutrition probably result in a greater susceptibility to STH owing to immunosuppressive changes being more frequent in these groups, while SAM on the other hand, antagonises STH survival through alterations in host gut physicochemical properties and the presence of nutritional deficiencies which do not favour STH growth and establishment [35]. Although we found no difference in helminth burden across our nutrition groups, these hypotheses suggest that depending on what degree of malnutrition is being investigated, varying differences in STH burden might exist among studies determining what role nutrition might have on the susceptibility to STH infections. With a similar study design to ours, the Zairean study documented a lower prevalence of STH infections in children with SAM. The SAM population in this study were all children with kwashiorkor (oedematous malnutrition), in contrast, with our study participants who all had marasmus. Kwashiorkor and marasmus are known to have different aetio-pathogenesis and clinical features [36]. It is therefore possible that they may also have different responses to STH infection, hence the observed difference in prevalence.

We did not find any difference in STH intensities between severely acutely malnourished and well-nourished participants, suggesting that malnutrition might also not predispose to higher intensities of soil-transmitted helminth infections in pre-school age children. Studies by Papier et al [18] and Oninla et al [32] have also compared the intensities of STH infection between malnourished and healthy children. While our results are similar to that of Oninla et al*,* [32] they contrast with those reported by Papier et al [18] who reported significantly higher intensities of STH in malnourished children. Our study and that of Oninla et al [32] used the WHO z-scoring system to classify nutritional status while Papier *et al* [18] used the recommended energy and nutrient intake (RENI) in the previous 24-h. The degree of nutritional deficiencies might thus vary between these studies and could account for the observed variation.

Overall, there was a low prevalence of STH infection among the under-five population within this study. Similar low STH prevalences have been described in some other similar settings [37]. Our findings however contrast with available local evidence of endemicity of STH infection [6, 22]. An explanation for this might be our study population. We recruited only pre-school children and the median age of our participants was 14 months. Previous evidences of the local endemicity of STH infection have been mainly from school-aged populations [22]. Pre-school aged children tend to have much lower STH prevalence than school-aged children [38, 39]. This might not be unrelated to school-aged children having greater dietary diversity as well as the propensity to explore their environments when compared to pre-school aged children. This ultimately predisposes them to a greater risk of acquiring these helminths from their surrounding environments. Another local study documented a higher prevalence than ours in pre-school children [26]. This study was however carried out more than a decade ago when widespread anti-helminthic chemoprophylaxis in pre-school children was not common practice in our setting. Our current findings of the low prevalence of STH among pre-school children in the study area has important local epidemiological implications. This is because the World Health Organisation recommends mass treatments in areas with prevalence greater than 20% [40]. In populations where endemicity is low, intensities of STH infection are usually low or moderate (as was documented in the current study) and are not associated with significant morbidities and as such, routine anti-helminthic prescriptions are not necessary [40]. In these instances, unwarranted anti-helminthic exposure would increase drug pressure and could possibly lead to anti-helminthic drug resistance. To forestall the occurrence of this, targeted anti-helminthic campaigns in such settings effective local monitoring programs would be necessary for the current community STH prevalence. These programs would need to be at sub-national and sub-regional levels, as the overall helminthic prevalence is usually not always reflective of internal region prevalence [4].

### Strengths and limitations

Our study recruited children with SAM from OTPs that served surrounding local communities. This allowed us recruit malnourished participants that were representative of the average child in the community with SAM. Our study improved on previous local studies that were conducted in hospital-based settings [33]. We also minimised detection bias during stool sample analysis as our laboratory research team was blinded to the nutritional arm of the study participants. Although both our nutrition groups differed in age distribution as the median age of SAM children was significantly lower than the well-nourished study population, the effect of age as a possible confounder in the results was however adjusted for using logistic regression analysis. The varying age distribution between our study populations could be explained by SAM being commoner in younger age groups and as such the majority of malnourished children attending the nutrition clinic were less than 2 years.

Our study participants where developmentally immature and as such could not produce on-the-spot stool specimen. We thus had to rely on parents bringing freshly voided stool specimens on scheduled follow-up visit. Because CMAM OTP centres serve close surrounding communities, we believe stool samples would not have been compromised before getting to us. Following the receipt of these specimens, we ensured samples were transported in ice boxes to the research lab where they were preserved and stored for analysis.

While we predetermined our study sample size calculations, setting the study power at 90% and using the STH prevalence data and the calculated effect size from a previous study [26], our documented prevalence of STH in both nutrition groups, were much lower than those used in estimating our sample size. As such, it is possible our study might have been underpowered to detect an existing difference in the prevalence or the intensity of STH between healthy children and children with SAM.

## Conclusions

The overall STH prevalence among pre-school children was low in Kano and we did not find the prevalence and the intensity of STH infection to differ significantly between preschool children with SAM and well-nourished children. Our findings confirm the World Health Organisation recommendation that at low levels of prevalence and intensity, interventions to control STH are not necessary.

## Abbreviations

AIC: Akaike’s Information Criteria
CMAM: Community Management for Acute Malnutrition
MAM: Moderate Acute Malnutrition
OTPs: Out-patient Therapeutic Programs
RUTF: Ready to Use Therapeutic Foods
SAM: Severe Acute Malnutrition
SSA: Sub-Saharan Africa
STH: Soil-transmitted helminth/ helminthic
UNICEF: United Nations Children’s Fund
WHO: World Health Organisation
